# Primary cilia biogenesis and associated retinal ciliopathies

**DOI:** 10.1016/j.semcdb.2020.07.013

**Published:** 2020-07-31

**Authors:** Holly Y. Chen, Ryan A. Kelley, Tiansen Li, Anand Swaroop

**Affiliations:** Neurobiology, Neurodegeneration and Repair Laboratory, National Eye Institute, National Institutes of Health, MSC0610, 6 Center Drive, Bethesda, MD 20892, USA

**Keywords:** Sensory cilia, Ciliogenesis, Photoreceptor, Retinal degeneration, CEP290, Intracellular transport

## Abstract

The primary cilium is a ubiquitous microtubule-based organelle that senses external environment and modulates diverse signaling pathways in different cell types and tissues. The cilium originates from the mother centriole through a complex set of cellular events requiring hundreds of distinct components. Aberrant ciliogenesis or ciliary transport leads to a broad spectrum of clinical entities with overlapping yet highly variable phenotypes, collectively called ciliopathies, which include sensory defects and syndromic disorders with multi-organ pathologies. For efficient light detection, photoreceptors in the retina elaborate a modified cilium known as the outer segment, which is packed with membranous discs enriched for components of the phototransduction machinery. Retinopathy phenotype involves dysfunction and/or degeneration of the light sensing photoreceptors and is highly penetrant in ciliopathies. This review will discuss primary cilia biogenesis and ciliopathies, with a focus on the retina, and the role of CP110-CEP290-CC2D2A network. We will also explore how recent technologies can advance our understanding of cilia biology and discuss new paradigms for developing potential therapies of retinal ciliopathies.

## 1. Introduction

The cilium is an evolutionarily conserved and ubiquitous microtubule-based organelle, which participates in a variety of cellular processes critical for development and homeostasis. Based on the ability to beat rhythmically to move extracellular fluid or lack thereof, the cilium can be classified as motile and non-motile. In vertebrates, motile cilia are mainly present in specialized cells, such as spermatozoa, epithelial lining of the respiratory tract, brain ventricles and the embryonic node [1,2]. Non-motile cilia, also called primary cilia, are largely associated with sensing extracellular chemical and mechanical cues and mediating signal transduction [3].

As one of the most ancient organelles observed, cilia were first documented by Van Leeuwenhoek in the 17th century for their motile properties in protozoan [4]. Contrary to the motile cilia with obvious beating dynamics, the non-motile primary cilia in metazoans, especially mammals, have long been considered vestigial until the discovery of the relationship between flagella/primary cilia biogenesis and polycystic kidney disease (PKD). Defects in the *tg737* gene, a homolog of *Chlamydomonas IFT88*, result in compromised primary cilium assembly in cells lining the urinary tract tubule, leading to mis-localization of PKD-related proteins [5,6]. Soon thereafter, primary cilia defects were shown to impede Sonic hedgehog (Shh) and calcium signaling [7,8], thereby implicating the primary cilium as a signaling organelle. Subsequent *in vivo* and *in vitro* studies demonstrated a major role of primary cilia in transducing exogenous cues and modulating numerous signaling pathways including Hedgehog (Hh), Wingless (Wnt), mammalian target of Rapamycin (mTOR), G protein-coupled receptors (GPCR), platelet-derived growth factor receptor (PDGFR)-alpha, transforming growth factor (TGF)-beta and Notch [9,10]. These studies have established primary cilia as the cellular antenna and a signaling hub for tissue morphogenesis and homeostasis [11]. Underscoring its importance, compromised biogenesis and/or dysfunction of the primary cilium has been identified in a broad spectrum of phenotypically overlapping yet highly variable disorders called ciliopathies, which can be associated with retinal degeneration, cystic renal disease, obesity, liver dysfunction, skeletal deformities, congenital heart defects, and brain developmental abnormalities [12,13]. Notably, among over 200 reported cilia-associated clinical manifestations in the Online Mendelian Inheritance in Man database (OMIM; https://omim.org), retinal degeneration is a highly penetrant phenotype and appears in over half of the ciliopathies.

**Fig. 1.**
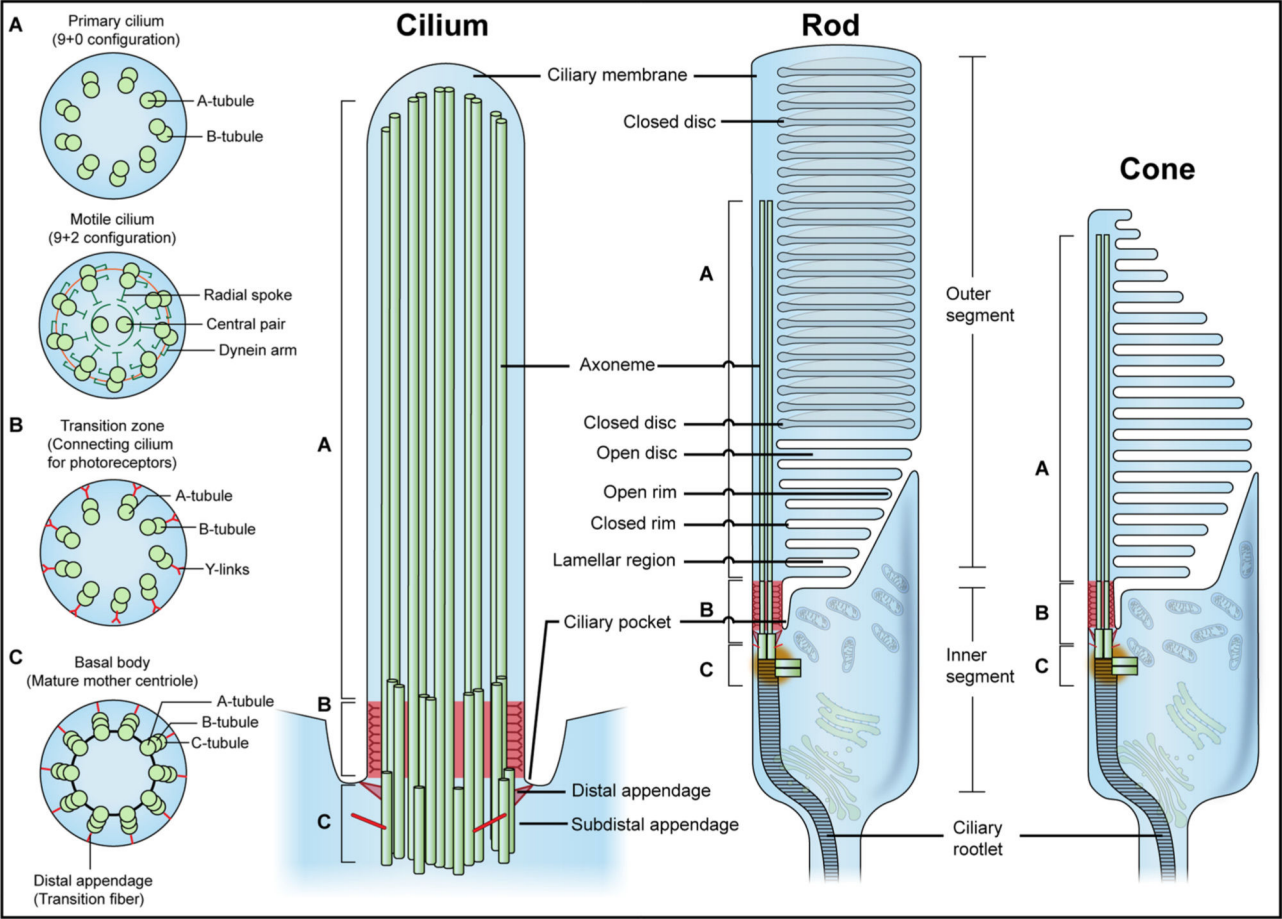
The architecture of the general primary cilium (left) and the photoreceptor outer segment (right). The primary cilium consists of a ciliary membrane and an axoneme. The ciliary membrane is continuous with the plasma membrane but differs in compositions to regulate diverse signaling pathways. In most mammalian cells, the plasma membrane invaginates at the base of the axoneme, forming the ciliary pocket for endocytosis and docking of intraflagellar transport particles. The axoneme elongates from the basal body (BB), which is the mature mother centriole with distal appendages and subdistal appendages. Cross-section diagrams at different positions of the primary cilium with a doublet (axoneme) (“9 + 0” configuration), doublet with Y-links (transition zone), and triplet microtubule structure (basal body) are shown in upper A, B, and C insets, respectively. In motile cilia, the axoneme displays “9 + 2” configuration, with an additional pair of microtubules in the center (Lower A inset). Photoreceptors feature a gradual doublet (base) to singlet (tip) microtubule transformation in the ciliary axoneme [125]. Photoreceptors harbor distinct features to accommodate their sensory function: the outer segment contains tightly packed discs with phototransduction machineries to efficiently capture photons; the inner segment possesses the endoplasmic reticulum, Golgi apparatus, and a large number of mitochondria to meet the high energy demand and biosynthetic needs of the photoreceptors; the ciliary rootlet anchors the BB to the inner segment to stabilize the outer segment.

The vertebrate retina is a light-sensitive tissue comprising six major types of neurons (rod and cone photoreceptors, horizontal, bipolar, amacrine and retinal ganglion cells) as well as one type of glial cell (Müller glia), which form a laminated structure interconnected by synapses for transmission of visual signals [14]. The photoreceptors are involved in capturing photons and converting the stimuli into electrical responses *via* a signaling cascade called phototransduction. The electrical signals are then integrated and processed by interneurons (bipolar, horizontal and amacrine cells) and transmitted to the brain through the optic nerve consisting of bundled axons of retinal ganglion cells [15]. Phototransduction is initiated in the modified cilium of photoreceptors called the outer segment (OS), which is structurally and functionally adapted for efficient photon capture. Rod and cone photoreceptors share a similar OS morphology and protein composition but differ in important details related to their distinct functions. Rods primarily function in dim light and are capable of detecting even a single photon, whereas cones are responsible for bright light and color vision [14] (Fig. 1). The OS is continually renewed, with new discs added at the base and older discs shed at the tip. The shed discs are then phagocytosed by retinal pigment epithelium (RPE) that is critical for maintaining photoreceptor function and survival [16,17].

Mutations in over 200 genes (RetNET, https://sph.uth.edu/retnet/) can lead to dysfunction and/or death of photoreceptors in retinal degenerative diseases including retinitis pigmentosa (RP) and Leber congenital amaurosis (LCA), which constitute a significant cause of incurable vision impairment or blindness worldwide. Many of these genes are photoreceptor- or RPE-specific, whereas others are expressed broadly but manifest an overt disease only in the retina. This review focuses on biogenesis of the primary cilium and on ciliopathies, especially those exhibiting retinal phenotypes. As an easily accessible tissue for *in vivo* studies, the retina is an excellent model to investigate complex features of cilia formation and ciliary transport, ciliopathies, and evaluation of treatment options.

In the following sections, we begin by describing the structure of the primary cilium, followed by the process of ciliogenesis. Next, we focus on the unique features of the modified cilium in photoreceptors and elaborate on genetic mutations and disruption in macromolecular complexes that contribute to retinal ciliopathies, using CP110-CEP290-CC2D2A network as an example. Finally, we conclude with recent advances in innovative technologies and therapies relevant to retinal ciliopathies.

## 2. Structure of the cilium

In most mammalian cells, the cilium is typically 250–300 nm in diameter and 1–10 μm in length [18,19]. The cilium consists of a microtubule-based backbone, called the axoneme, and a ciliary membrane continuous with the plasma membrane [20] (Fig. 1). The axoneme elongates from the basal body (BB), which originates from the mature mother centriole (MC) with a structure of 9 distal appendages (DA) and varying numbers of subdistal appendages (SDA) [21] (Fig. 1C). As in centrioles, the BB is composed of a barrel shaped, 9-fold radially symmetric structure with triplet microtubules [22]. Its proximal end is anchored by striated ciliary rootlets, which are composed of polymers of intermediate filament-like protein rootletin, to provide structural support to the cilium [23]. The distal end of the BB features a transition from triplet microtubules to doublets of ciliary axoneme. This transition zone (TZ) in the proximal portion of the cilium possesses a unique functional significance and features Y-shaped linkers that tether the ciliary axoneme to the ciliary membrane [24] (see Fig. 1B). In photoreceptors, the TZ is commonly referred to as the connecting cilium (CC).

The ciliary axoneme emerges from the BB and is comprised of nine doublet microtubules known as outer doublets [25], which are subjected to post-translational modifications including acetylation, glutamylation, detyrosination and glycylation that are crucial for ciliary length, stability, functions and motility [20,26]. Other structural components include tektins and the protofilament ribbon proteins, which provide additional stability to axonemal microtubules [20]. To enable the bending motion, the axoneme of the motile cilium possesses an additional central pair of microtubules, which connect with the outer doublets by radial spokes and dynein arms (Fig. 1A). Thus, cross-sectional views of motile cilia usually present a 9 + 2 configuration. In contrast, primary cilium typically lacks the central pair of microtubules and contains a 9 + 0 configuration [27] (see Fig. 1A). Exceptions to this general rule include nodal cilia, which are motile but lack the central pair of microtubules [28]. Some sensory cilia, such as kinocilia of cochlear hair cells and olfactory sensory neurons, while immotile, do possess the central pair of microtubules [29,30]. In most mammalian cells, an invaginated plasma membrane, known as the ciliary pocket at the base of axoneme, is the site of active endocytosis and docking of intraflagellar transport (IFT) particles [31].

## 3. Primary cilia biogenesis

**Fig. 2.**
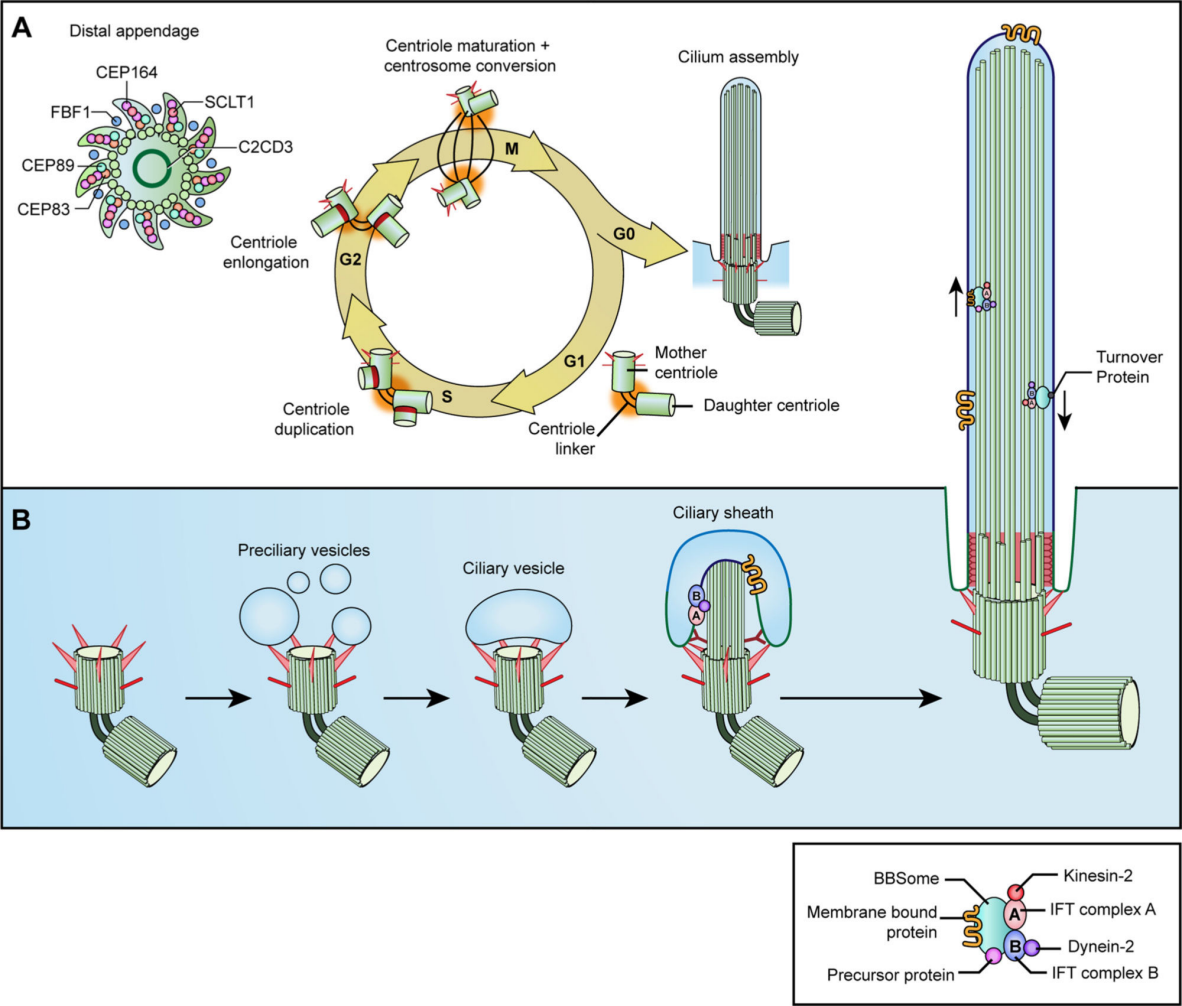
Centriole and cilium biogenesis. (A) Regulation of centriole biogenesis during the cell cycle. In the G1/S phase of proliferating cells, assembly of new centrioles are initiated on both the mother centriole and the daughter centriole, creating two mother-daughter centriole pairs. The newly formed centrioles elongate throughout the G2 phase. In the late G2 phase, the daughter centriole from the previous cell cycle acquires distal appendages and subdistal appendages by sequential recruitment of their structural components. Subdistal appendages anchor microtubules and facilitate the formation of the pericentriolar material. To initiate the M phase, the two pairs of centrosomes separate, migrate to the opposite poles of the cells and establish bipolar spindles. Upon exit from cell cycle, the mother centriole docks to the plasma membrane by distal appendages for cilium assembly in response to specific developmental and/or environmental signals. (B) Intracellular pathway of cilium biogenesis. Ciliogenesis is initiated by docking of preciliary vesicles from the Golgi apparatus and recycling endosomes to distal appendages. These vesicles subsequently merge to form a large ciliary vesicle containing machineries for the maturation of the mother centriole and the trafficking of nascent cilia. Upon CP110 removal, the intraflagellar transport (IFT) complexes (IFT-A, pink oval and IFT-B, blue oval) and motor proteins (kinesin-2 motors, red ball and dynein-2 motors, purple ball) are recruited to distal appendages. The transition zone emerges shortly after the recruitment of IFT machineries and is characteristic of the Y-links. The ciliary axoneme elongates and the ciliary membrane extends with the transport of ciliary proteins and building blocks, forming the ciliary sheath. Fusion of the ciliary sheath with the plasma membrane exposes the primary cilia to the external environment.

Biogenesis of the primary cilium is a highly complex yet well-orchestrated process involving multiple cellular machineries and signaling pathways (Table 1 and Fig. 2). Formation of the cilium is initiated by apical migration of the MC to become the BB, followed by extension of the axonemal microtubules, formation of the TZ, and growth of the cilium through ciliary trafficking machineries such as IFT. Depending upon whether the elongation initiates at the cell surface or within the cytoplasm, the process of primary cilia biogenesis can be categorized as an extracellular or intracellular pathway, respectively [32]. The extracellular pathway is observed in polarized epithelial cells of the kidney and lung, whereas the intracellular assembly is adopted by most cells including photoreceptors [33,34]. We will primarily discuss the intracellular pathway in this review.

### 3.1. Mother centriole maturation

In mammalian cells, the centriole is an evolutionally conserved microtubule-based organelle with typical dimensions of ~200 nm in diameter and ~500 nm in length and a cylindrical structure of nine microtubule triplets [35]. A pair of perpendicularly positioned centrioles (the mother and daughter centrioles) recruit an amorphous proteinaceous meshwork, called the pericentriolar material (PCM), to form the centrosome [36]. The centrosomes are formed through interaction between the centrioles and less well-defined PCM proteins such as the γ-tubulin ring complex (γTuRC) and PCM1 [37,38]. As the primary microtubule-organizing center (MTOC) of the cell, the centrosomes are responsible for nucleation of microtubule assembly in interphase cells and docking of the bipolar microtubule spindle during mitosis [39]. The centrosomes can also act as the actin-organizing center in cells, in addition to its essential function in the regulation of cilium biogenesis and maintenance [34,40].

Centrosome duplication and conversion of the MC to the BB are tightly coordinated with the cell cycle. In the G1/S phase of proliferating cells, the daughter centriole (DC) loses the DC-enriched proteins, a process required for centriole duplication and maturation [34] (Table 1). Assembly of new centrioles is initiated at the proximal end of both the MC and the DC (Fig. 2A). In vertebrates, the centriole receptor CEP152 and CEP192, which encircle the proximal end of the template centrioles, recruit PLK4 and subsequently STIL [41,42]. Phosphorylation of STIL by PLK4 triggers the binding of SAS6 and initiates the assembly of the cartwheel, the structural base of the newly formed DCs, limiting the accumulation of PLK4 at the centrioles to prevent the formation of additional centrioles [43,44]. Facilitated by CEP135, SAS6 can oligomerize into a 9-fold symmetrical structure of the cartwheel [45]. Growth of the cartwheel depends on the addition of SAS6 to the proximal end of the stack to stabilize the structure [44]. The newly formed DCs remain tightly anchored to the side of the MC until centriole disengagement at the late mitosis or early G1 phase in the next cell cycle, a process catalyzed by a cysteine protease, called separase, and PLK1 [46,47] (Fig. 2A). This is a key licensing step that restricts centrosome duplication to occur only once during a cell cycle.

In the late G2 phase, the DC from the previous cell cycle begins to mature, acquiring DA and SDA structures by sequential recruitment of respective proteins to the distal end of the centriole [34] (Table 1) (Fig. 2A). DAs are responsible for membrane docking and recruitment of IFT machinery at the distal end [48]. As shown by super-resolution microscopy, DAs display a cone-shaped structure with the backbone of a pinwheel complex that is sequentially formed by CEP83, CEP89, and SCLT1 [49] (Fig. 2A inset). In turn, this complex recruits CEP164 and FBF1, the latter one demarcates the distal end of the DA matrix near the ciliary membrane [49] (see Fig. 2A inset). SDAs anchor microtubules and participate in endosome recycling [50]. Their formation is regulated by a different set of proteins including γ-tubulin, which directly participates in the nucleation of microtubules [34] (Table 1). CC2D2A initiates the hierarchical assembly of other SDA proteins by recruiting ODF2, which serves as the anchor for binding to CCDC120 and CCDC68, followed by the recruitment of CEP170 and Ninein [51].

To initiate the M phase, the two centrosomes, each with newly formed mother-daughter centriole pairs, segregate to the opposite poles of the cell and establish a bipolar spindle. Upon exit from the cell cycle, the MC gains competence for ciliogenesis in response to specific developmental and/or environmental signals (Fig. 2A).

### 3.2. Formation of ciliary vesicles (CVs) and the TZ

Docking of preciliary vesicles (PCVs) at DAs of the MC constitutes an initial step of ciliogenesis. PCVs originate as small cytoplasmic vesicles from the Golgi apparatus and the recycling endosomes and are transported to DAs through sequential actions of dynein, myosin MYO5A, and actin network (Fig. 2B). Dynein facilitates the transport of MYO5A-associated PCVs to the pericentrosomal area along microtubules, followed by subsequent delivery of these PCVs to DAs as mediated by MYO5A along the ARP2/3-associated branched actin network [52] (Fig. 2B). These PCVs fuse to form a large CV through membrane tubulation mediated by the ESP15 homology domain (EHD)-1, a SNARE membrane fusion regulator, and EHD-1 binding protein SNAP29 and EHD-3 [53] (Fig. 2B). Small GTPase ARL13B, F-BAR domain PACSIN proteins and components of the RAB8-RAB11 GTPase cascade, which are important for the transition of the MC to the BB, are enriched in the CVs [54,55]. RAB11 and microtubule-associated protein (MAP) 11 recruit RABIN8 (a guanine nucleotide exchange factor) to the CVs, a process mediated by transport particle protein (TRAPP) II complex [55,56]. RABIN8 then interacts with CEP164 to activate RAB8, which together with ARL13b promotes the growth of the ciliary membrane and selective trafficking of ciliary proteins to the nascent cilium [57–59].

In addition to docking of CVs and initiation of ciliogenesis, DA proteins also initiate elongation of the ciliary axoneme. Centriolar coiled-coil protein of 110 kDa (CP110) is localized to the distal end of the mature MC and “caps” the growing microtubules to modulate ciliogenesis [60,61]. Removal of CP110 is driven by a small calcium-binding protein CETN2 and recruitment of tau-tubulin kinase TTBK2 by DA protein CEP164, which subsequently phosphorylates various substrates including CEP164 itself, the kinesin KIF2A and CEP83 [62,63]. Concurrently with CP110 removal, the IFT machinery is recruited to the DAs by DA proteins and mediates the recruitment of ciliary motor proteins kinesin-2 and dynein-2, IFT proteins, and trafficking of ciliary axonemal components such as tubulin [53,64] (Fig. 2B). INPP5E, a lipid-modified phosphoinositide phosphatase involved in initiation of ciliogenesis and ciliary trafficking, is recruited to the ciliary base by CEP164 [65,66].

The TZ emerges shortly after IFT machineries are recruited to the DAs and is marked by the Y-links visible on cross sectional profiles (Fig. 2B). The Y-links are characterized by Y-shaped fibers tethering each microtubule doublet to the ciliary membrane and may be correlated to bead-like intramembranous particles, called ciliary necklace, that is visible from the exterior surface [67,68]. The Y-links and ciliary necklace exhibit a close connection with each other, yet their relationship and function(s) remain unclear [24,69]. Interactome analyses of the TZ proteins have identified two biochemically distinct TZ protein complexes. The NPHP complex is associated with nephronophthisis, whereas the MKS complex is abnormal primarily in Meckel (MKS) and Joubert syndrome (JS) and related disorders (JSRD) [24,70] (Table 1).

The NPHP and MKS complexes interact with each other through a network of loose connections, forming two major hubs: CEP290 in the MKS complex, which binds to NPHP5 of the NPHP complex, and Inversin (also called NPHP2) complex in the NPHP module, comprising of NPHP3, NEK8, ANKS6 and ANKS3 [65]. Other proteins and lipid components in the TZ are also believed to contribute to the unique composition and function of the TZ. For example, a ring-like structure consisting of the GTP-binding proteins of SEPTIN family (SEPTIN2, SEPTIN 7 and possibly other members) constitutes the diffusion barrier of the TZ [71]. Another example is phosphoinositide lipid PI(4,5)P_2_, which is restricted to the proximal ciliary membrane by the ciliary enzyme INPP5E and regulates TZ function [72]. PI(4,5)P_2_ also participates in the TZ maturation in *Drosophila* [73]. Additionally, a proteomic analysis of *Chlamydomonas* TZ uncovered a crucial role of ESCRT protein complex in shedding of extracellular vesicles from the primary cilium [74]. The corresponding TZ components of other species and their functions in primary cilia of mammals require further investigation. Super-resolution microscopy technologies demonstrate that the NPHP complex is close to the TZ microtubules, consistent with the tubulin-binding capacity of NPHP1 and NPHP4, whereas the MKS complex is mostly associated with the ciliary membrane [65]. However, the location of the protein complex hub CEP290 from different species is still controversial [65]. It is unclear whether it is species-specific, or cilia-type-specific, or related to the large size of CEP290.

TZ appears to be a hotspot for ciliopathies due to its complex protein networks and crucial roles in cilia biogenesis and functions. In mice, defects in the MKS complex cause more severe diseases and even embryonic lethality, whereas mutations in the NPHP proteins broadly lead to kidney defects along with occasional extrarenal phenotypes including photoreceptor degeneration [13] (Table 1). Curiously, mutations in RPGRIP1L in the NPHP complex can be associated with more severe ciliopathies such as Meckel-Gruber syndrome and JS and/or modify retinal degeneration phenotype in ciliopathies [75–77]. Ciliogenesis defects are more severe when both an NPHP and an MKS complex component are disrupted compared to when either one is impaired, suggesting some functional redundancy between the MKS and the NPHP complexes [65]. More severe defects can be caused by disruption in interaction between TZ complexes and IFT machineries [65]. How TZ complexes interact with IFT components and their role in ciliogenesis are still poorly understood.

After the formation of the TZ, the axonemal microtubules elongate with transport of ciliary proteins and building blocks of the ciliary axoneme such as tubulin (Fig. 2B). Fusion of the CV with the plasma membrane exposes the primary cilium to the external environment. The outer region of the ciliary vesicle then becomes a ciliary pocket adjacent to the ciliary membrane [18] (Fig. 2B).

### 3.3. Ciliary trafficking

Primary cilia have a distinct protein composition that can be attributed to a specialized ciliary trafficking machinery. Soluble proteins that are not associated with the membrane can enter the cilium by passive diffusion or active transport utilizing microtubule motor proteins. Passive diffusion of soluble proteins is size-dependent because of size-exclusion characteristics of the DA and the TZ. DA proteins are also referred to as transition fibers (TFs) when associated with the ciliary membrane or its precursors. Super-resolution microscopy of cross sections of TFs indicate ~60 nm space between adjacent ones [78], which may execute size-dependent entry and exit of soluble proteins in and out of cilia as part of a gating mechanism. A similar mechanism might be utilized by the TZ, in which the Y-links and the protein meshwork between them have been proposed to act as a molecular sieve-like barrier [79]. Therefore, the capacity of a protein moving in and out of the cilia might depend on size in passive diffusion.

**Fig. 3.**
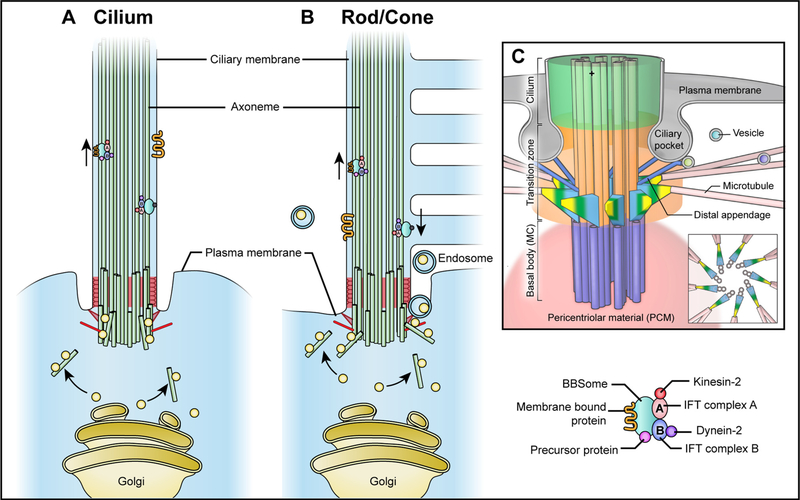
Intraflagellar transport (IFT) of the general primary cilium (A) and photoreceptor outer segments (B). A schematic of the base of primary cilium is shown at (C). The IFT machineries are composed of the microtubule motors (kinesins and dyneins), IFT complex (A and B) and accessory proteins (*e.g*. TULP3, the BBSome). Most ciliary proteins are trafficked to the base of the primary cilia from the post-Golgi network through microtubules in vesicles with the accessory proteins (green oval), which serve as membrane adaptors for specific cargo proteins and IFT complexes. Complex A (pink oval) and complex B (blue oval) move along the microtubule together, yet they have distinct biochemical constituents and functions. Complex B interacts with plus end-directed kinesin-2 motors (red ball) and participates in anterograde transport from the ciliary base to the tip, which is essential for cilia assembly and maintenance. Complex A binds to a minus end-directed motor cytoplasmic dynein-2 (purple ball), which is responsible for retrograde IFT to move cargo proteins from the ciliary tip to the base. In photoreceptors with constant and rapid renewal of the outer segment, besides the conventional pathway by IFT machineries, highly enriched phototransduction proteins (*e.g.* rhodopsin) can also be transported through recycling endosomes.

Active transport would circumvent the limitation of protein size, relying on cargo association with IFT machineries, which is the primary route for soluble and membrane-associated ciliary proteins that are transported in and out of the cilium. The IFT machineries are composed of microtubule motors, IFT complexes and accessory proteins [20] (Fig. 3A). Depending on the direction of IFT, the movement of cargo proteins along the axoneme is catalyzed by two different sets of motor proteins: kinesins and dyneins. Anterograde IFT, which transports cargos from the ciliary base to the tip, is executed by plus end-directed kinesin-2 motors (Table 1). Cytoplasmic dynein-2 is a minus end-directed motor responsible for retrograde IFT, which moves cargo proteins from the ciliary tip to the base [80] (Table 1).

The two IFT complexes, IFT complex A and complex B, include components that are highly enriched in protein-protein interaction domains, which enable them to carry substantial cargo proteins. Yet, complex A and complex B have distinct biochemical constituents and functions. Complex B participates in anterograde transport, which is essential for cilia assembly and maintenance [20], whereas complex A does not seem to be required for cilia assembly but is essential for retrograde transport [20] (Table 1). Although participating in IFT of different directions, complex A and B move together along the ciliary axoneme. Separation of complex A and B leads to defective ciliary transport, suggesting cooperative interaction between the IFT complexes [81].

IFT accessory proteins such as KAP act as membrane adaptors for specific cargo proteins and IFT complexes [82]. Some of these accessory proteins are also essential for the architecture of IFT complexes [83]. To access the cilium, cytosolic proteins interact with microtubule motors, IFT complexes and cargo adaptors such as TULP3 and the BBSome (Table 1). Integral membrane-associated cargo proteins need ciliary localization sequences (CLSs) to interact with the IFT machineries. For example, a specific group of GPCRs is transported into the cilium by IFT accessory proteins such as TULP3, which serves as a membrane adaptor in complex A [84]. Ciliary localization of lipid-anchored proteins such as RP2 requires binding to an adaptor importin- β2, which associates with the KIF17 component of the kinesin-2 motor complex [85]. Peripheral membrane proteins can also be transiently solubilized by interaction with lipid-binding transporter proteins to cross the ciliary gate. Two important examples are UNC119 and PDE6D, which recognize the myristoylated and prenylated residues of targeted cargo proteins, respectively [65]. Binding of UNC119 and PDE6D to targeted residues inhibits association of cargo proteins to the plasma membrane for trafficking across the TZ. Once inside the cilium, a small GTP-binding protein ARL3 binds to transporter proteins, thereby inducing the dissociation of transporter-cargo complex and enabling the cargo to associate with the ciliary membrane. ARL3 activity depends on RP2 and ARL13B, which regulate the association with GDP and GTP [66,86]. These two trafficking systems antagonize each other, probably since both UNC119 and PDE6D rely on the activity of ARL3 [87].

The IFT machineries are also needed to transport membrane-associated proteins out of the cilium. GPCRs are transported out of cilia by another set of adaptors: the small Arf-like GTPase ARL6 (membrane-associated with GTP and the BBSome bound; BBS3), and the sole effector of ARL6 (BBS3) – the BBSome [88]. The BBSome acts as an IFT cargo to mediate the exit of other proteins from cilia such as Hh mediator, GPR161 [89].

Ciliary and nuclear transport share several interesting similarities. First, both nuclear and ciliary import require specific localization signals, although so far no clear consensus sequence has been identified for ciliary localization or protein recognition machineries [90]. Second, the TZ has a similar selective protein transport function as the nuclear pore, which allows cargo movement between the nucleus and the cytoplasm [91]. Third, Ran-GTP in the cilium and Ran-GDP in the cytoplasm are important for ciliary transport, as is in nuclear transport [92,93]. Fourth, numerous ciliary proteins have additional functions in the nucleus to regulate gene expression during the cell cycle or when the cell is under stress. For example, CEP290, which is hub of the TZ, also localizes to the nucleus and participates in the cellular response to DNA damage and replication stress [94]. CEP290 interaction partner RPGR, together with RPGRIP1, might have a role in recruiting nucleophosmin to the centrosome in the regulation of cell cycle [95]. For more information about the nuclear functions of ciliary proteins, we direct readers to other excellent reviews [96,97].

### 3.4. Cilia disassembly

The primary cilium is resorbed or shed before cells re-enter the cell cycle. Cilium disassembly requires depolymerization of axonemal microtubules and scission of the ciliary membrane. One of the key regulators in this process is the mitotic kinase AURORA A, which is activated upon cell cycle re-entry. Activated AURORA A phosphorylates and stimulates histone deacetylase HDAC6, which then destabilizes and depolymerizes axonemal microtubules [98,99]. Another histone deacetylase HDAC2 positively regulates the expression of AURORA A to promote cilium disassembly, which can lead to development of various types of cancers when misregulated [100]. The kinesins KIF2A and KIF24 also participate in this process. Located at the proximal side of the BB, KIF2A is degraded and KIF24 (which is associated with CP110) is inhibited upon ciliogenesis [101]. Upon re-entry to the cell cycle, KIF2A is activated by G2/M phase kinase PLK1 and promotes microtubule depolymerization. Similarly, KIF24 is phosphorylated and activated by NEK2, a cell cycle kinase expressed between S and G2 phase, to promote microtubule disassembly. This process is irreversible once S phase begins [102,103].

Cilium disassembly also requires remodeling of the ciliary membrane, which mainly involves the ciliary pocket and scission of the distal tip of the cilium. The ciliary pocket is an actin-docking site located at the periciliary subdomain at the junction of ciliary and plasma membranes [104]. One important mediator between mitogenic stimulation and cilium disassembly is the insulin-like growth factor 1 (IGF1) signaling pathway. Upon activation of the IGF1 pathway by binding of IGF1 to ciliary IGF1R, phosphorylated DYNLT1 is recruited to the TZ [105]. DYNLT1 interacts with F-actin and other active polymerization regulators to remodel the ciliary pocket and enhance clathrin-mediated endocytosis. In addition, scission of the cilium from the distal tip can be observed upon growth stimulation. The site of cilium decapitation is determined by ciliary distribution of PI(4,5)*P*_*2*_, which induces actin polymerization in coordination with other actin regulators [106]. AURORA A depletes ciliary INPPE5 and re-distributes PI(4,5)*P*_*2*_ to facilitate actin nucleation and CV release [107]. Notably, CV release preferentially removes the IFT-B complex, which participates in anterograde transport for cilium growth, thereby promoting cilium disassembly [106]. The ubiquitin-proteasome system components also participate in the cilium disassembly [108,109]. For more details, we refer readers to reviews that cover this topic more extensively [34,110].

### 3.5. Regulation of cilia assembly/disassembly

The primary cilium is disassembled before mitosis and reassembled after mitotic exit or upon mitogen deprivation, highlighting a close coordination between the cell cycle and ciliogenesis. As such, the assembly and disassembly are tightly regulated processes. The timing of cilium assembly is controlled by transcriptional regulation as well as growth factor/mitogen stimulation. For example, the RFX family transcription factors are reported to control the expression of BB machineries and cilia-specific proteins required for ciliogenesis [111].

A number of growth factors/mitogens are involved in regulating ciliogenesis and cell cycle. For instance, serum deprivation stimulates ciliogenesis in cultured cells. Serum lysophosphatidic acid inhibits cilium development *via* the downstream PI3K/AKT signaling pathway, which regulates RABIN 8 preciliary trafficking and thus the initiation of RAB8-dependent ciliogenesis by the RAB11-RAB8 cascade [112]. Epidermal growth factor (EGF) and platelet-derived growth factor (PDGF) both suppress ciliogenesis and initiate cilium disassembly utilizing NEK2 and AURORA A. In addition, binding of PDGF to PDGF receptor β activates PLCγ, which releases intracellular Ca^2+^ from the endoplasmic reticulum to activate calmodulin and AURORA A [113,114].

Although primary cilia are microtubule-based organelles, a crucial role of actin has been suggested in the regulation of their positioning, assembly, and functions through branched F-actin and the actin nucleating ARP2/3 complex. The latter interacts with the nucleation promoting factor WASH, which is recruited to the centrosome by PCM1 [115]. Therefore, the centrosome likely functions both as a microtubule- and actin-organizing center. Cytoplasmic and cortical branched F-actin networks mainly have an inhibitory role in cilium assembly, lengthening and material exchange with the cytoplasm. Cytoplasmic F-actin inhibits the transport of ARL13B-associated ciliary vesicles to the BB, whereas those at the base of cilia and ciliary pocket act as a barrier for the diffusion of plasma membrane proteins such as GPCRs [88,116]. Inside the cilium, F-actin organizes lipid corrals to limit receptor diffusion and regulates its length by decapitation at the distal tip [106,117]. WASH activates ARP2/3 to nucleate actin polymerization at the centrosome to form pericentrosomal networks, which are required for the trafficking of MYO5A to the BB and the formation of a diffusion barrier to regulate passage into and out of the cilium [40]. FHDC1, a unique member of the formin family, coordinates actin and microtubule dynamics at the SDA to anchor BB positioning and regulate cilia length [118]. ARP2/3 activity may also coordinate with lysine deacetylase KDM3A to modulate axonemal microtubule assembly by controlling IFT entry [119,120]. For more details on actin-based regulation of ciliogenesis, see a recent review [40].

## 4. Specialized sensory cilia of photoreceptors

Photoreceptors are highly specialized neurons that are anatomically organized into a distinct layer in the retina. As mentioned earlier, the OS of a photoreceptor is a modified sensory cilium containing phototransduction components, whereas the inner segment (IS) houses the endoplasmic reticulum, Golgi apparatus, and mitochondria to meet the high energy demand and biosynthetic needs of the cell. The presynaptic terminal of a photoreceptor connects to the second-order neurons including bipolar and horizonal cells [121]. The visual process is initiated by photon capture in the OS and the phototransduction cascade ensues. A comprehensive proteomic study of mouse photoreceptor OS and connecting cilium (the TZ) identified ~2000 proteins, of which only a few hundred are shared with the primary cilium of other cell types, as the OS contains abundant specific proteins subserving phototransduction [122].

### 4.1. Morphology of the OS

The OS of rod and cone photoreceptors is largely homologous in structure but differs in morphology and protein compositions. While the rod OS is cylindrical, the cone OS is tapered and conical in most vertebrate species including humans (see Fig. 1). The OS of both photoreceptors possesses a similar cytoskeletal backbone when compared to the primary cilium of other cell types. The ciliary rootlet is more highly developed than any other cell types and anchors the proximal end of the BB and extends deep into the IS to stabilize the OS [123]. Axonemal microtubule doublets extend distally, reaching about one half of the rod photoreceptor OS and to the tip in the cones [124]. As the microtubules extend distally, the doublets gradually transition to singlets [125]. The Y-links crosslink microtubules to the surrounding membrane at the CC [126]. The CC measures ~0.3 μm in diameter and ~1–1.5 μm in length in both rods and cones in most vertebrate species [127], which is almost 3 times the length of the TZ of other primary cilia. Recent advances in super resolution microscopy have uncovered a specialized feature of the photoreceptor CC, in which the proximal portion is homologous to the TZ of primary cilia in other cell types, whereas the distal region is a photoreceptor-specific extension. The specialized distal zone is maintained by SPATA7 and its interacting partners, such as RPGR and RPGRIP1, which have important functions in stabilizing axonemal microtubules [128]. Molecular mechanisms underlying the maintenance of the ciliary axoneme by the distal CC and why it is specialized in photoreceptors require further investigation.

Photoreceptor OS is among the largest of mammalian cilia to accommodate tightly stacked membranous discs harboring visual pigments and other phototransduction proteins [129]. In mouse rods, membranous discs are arranged at a density of ~30 discs per μm totaling up to 2000 discs. These discs are densely packed with visual pigments and associated proteins for efficient photon capture and rapid signal transduction [130]. While rod discs are enclosed except for the nascent discs at the base, cones largely maintain open discs through the entire OS [131–133] (see Fig. 1). The rod visual pigment rhodopsin, the most abundant disc membrane protein in rod cells, is organized as rows of dimers to reach a density of ~48,000 monomer per μm^2^ to provide additional structural support and maintains the disc configuration [134].

Another unique feature of the photoreceptor OS is its continuous renewal. Each day ~10% of the OS is shed from the distal tip and phagocytosed by the RPE. The length of the OS remains constant as new discs are formed at the base [135,136]. Rapid renewal of the OS requires efficient generation of new discs. Formation of new discs starts with evagination of the OS membrane driven by an expanding branched actin network, forming open discs consisting of closed rim, open rim and lamellar regions [137,138] (see Fig. 1). PRPH2 and its homolog ROM1 both form homodimers, which further associate to form tetrameric complexes that are located exclusively at the disc rim [139]. These complexes are required for the maintenance of hairpin-like curvature of the closed rim structure and morphogenesis of discs, which would otherwise be excreted as ectosomes [140]. As the newly forming discs protrude from the plasma membrane and expand, they are maintained flat by PRCD, without which the nascent discs bulge and release extracellular vesicles [141]. In rod photoreceptors, the closed discs are formed once a disc reaches the full OS diameter and the expansion of an open rim encloses the space between adjacent surfaces of the two lamellae [133]. An integral membrane protein CD113 localizes to open rims and forms a connection with the IS plasma membrane in the ciliary pocket *via* PROM1 and PCDH21 [142,143], suggesting a plausible role of the ciliary pocket in biogenesis of OS discs. The ciliary pocket and the CC are connected through other filamentous proteins, mainly the USHER protein complexes, for the maintenance and stability of the OS [144,145]. The ciliary pocket is also a site for docking of post-Golgi vesicles and transport of OS components such as opsins, an alternative approach of ciliary protein transport in photoreceptors [144,146].

### 4.2. Protein transport to OS

In addition to basic building blocks of primary cilia, additional photoreceptor-specific proteins are required for the OS to mediate phototransduction. Together with the rapid renewal, these features of photoreceptor OS necessitate robust protein synthesis, high energy supply in the IS, and efficient transport of OS-bound proteins. Similar to primary cilia of other cell types, ciliary gating of the OS is established by hydrogel-like size-excluding barriers of transition fibers, which only allow the passage of proteins less than 70 kDa, and the protein meshwork in the CC [147]. The selective trafficking of microtubule components for the ciliary axoneme in photoreceptors is analogous to that of the primary cilium in other cell types. Protein cargos are delivered from the base to the tip by anterograde transport, which is mediated by IFT-B complex with kinesin-2 motor complexes. Once the cargos are delivered, the IFT-B components and other ciliary proteins are transported from the tip to the base by dynein-2-driven IFT-A complexes *via* retrograde transport [148] (Fig. 3B).

OS-bound membrane proteins appear to be transported through a somewhat different route (Fig. 3B and C). Rhodopsin is the most abundant protein in rod OS, comprising more than 80% of the proteins in the OS [149]. The transport of rhodopsin to the OS is likely through a conventional pathway, which is accomplished through fusion of carrier vesicles with the periciliary membrane, and an unconventional pathway, which is achieved by recycling endosomes. In the conventional pathway, rhodopsin is synthesized in the endoplasmic reticulum and transported through the Golgi and *trans*-Golgi network to sort into carrier vesicles targeting the OS. This process is mediated through specific ciliary localization signals at the C-terminus of rhodopsin to facilitate the interaction with ciliary targeting molecules and fusion with carrier vesicles [150]. Rhodopsin is transported through the CC to the site of disk morphogenesis by two motor proteins kinesin-2 and myosin-7a, the latter of which is an actin-dependent molecular motor localized to the periciliary membrane complex in mouse and calyceal processes in primates [145,151]. The C-terminus of rhodopsin also interacts with SARA, an early endosome protein, suggesting the feasibility of rhodopsin transport in an unconventional pathway by early and recycling endosomes [152,153]. In addition, RAB11 also regulates the sorting of rhodopsin at recycling endosomes in cultured MDCK cells [154]. Whether rhodopsin is transported to the OS through either or both of these pathways requires further investigation. Other membrane-associated proteins, such as PRPH2, utilize both conventional and unconventional pathways to reach the ciliary membrane and disc rim region [155,156].

Photoreceptors feature light-activated translocation of soluble phototransduction proteins including transducin, arrestin and recoverin. In the dark, translocation of transducin into the rod OS may increase the gain of phototransduction, while under the light, translocation of arrestin to the OS could accelerate inactivation of phosphorylated rhodopsin and expedite recovery of the photoresponse [157]. Diffusion of these proteins is energy independent but requires steric volume exclusion, suggesting a steric interaction between soluble translocated proteins and highly constrained space between OS disc membranes [158].

## 5. CP110-CEP290-CC2D2A network in early ciliogenesis and ciliopathies

Retinal ciliopathies can be caused by mutations in genes for photoreceptor OS structure and/or function. Notably, mutations in the same gene can lead to both non-syndromic and syndromic retinal ciliopathies based on the type and the location of mutations as well as individual modifier genetic variations. In this section, we mainly focus on the CP110-CEP290-CC2D2A network in early ciliogenesis and ciliopathies.

### 5.1. CP110

CP110 and its interaction partner CEP97 are critical for cell cycle regulation and were the first proteins shown to negatively regulate cilia assembly [63]. CEP97 is a Ca^2+^-binding protein that likely serves as a chaperone to stabilize CP110 for recruitment to the distal centriole [63,159]. CP110 forms large complexes with calmodulin and centrins, which constitute a family of calcium-binding phosphoproteins in the centrosome of eukaryotic cells [160]. CETN2 was reported to regulate CP110 levels and ciliogenesis in chicken but does not seem to impact the development of photoreceptor OS in mouse [161].

CP110 levels and localization to the centrosome are tightly regulated in a cell cycle-dependent manner. CP110 protein amount drops significantly in the G2/M and G0/G1 phases as a consequence of transcriptional controls, ubiquitin-mediated proteasomal destruction, and microRNA-mediated turnover of CP110 mRNA [162–164]. Knockdown of either CP110 or CEP97 leads to inappropriate cilium formation in proliferating cells and genome instability, whereas overexpression of either inhibits ciliogenesis in non-proliferating cells [160]. CP110 caps the distal end of centrioles but is absent at the BB in ciliated cells. The removal of CP110 from the distal MC is associated with two serine/threonine kinases, TTBK2 and MARK2. Recruitment of TTBK2 relies on DA components and MARK2 interacts with SDA component ODF2, indicating crucial roles of DA and SDA components in the removal of CP110 from the MC to promote cilium assembly. In addition to CEP97, CP110 interacts with other proteins that further control its activity and consequently cilia formation. For example, CEP104 is a microtubule plus-end tracking protein that co-localizes with CP110 at the distal end of centrioles in proliferating cells and is absent from the BB during ciliogenesis. However, CEP104 counteracts the effects of CP110 and CEP96 and promotes the onset of ciliary axoneme growth [165]. CP110 and CEP290 also bind to RAB8A for cilium assembly [166]. Another interaction partner of CP110 is KIF24, which binds to CP110 and CEP97 to stabilize the complex and inhibit ciliogenesis [103].

The insights on CP110 function are primarily based on cell culture studies; nonetheless, animal models have provided significant new information on CP110 function *in vivo*. Elevated levels of CP110 are associated with chronic rhinosinusitis, a respiratory disease with abnormal or lack of motile cilia, consistent with cell culture data [167]. Studies using the *Cp110*-knockout mice suggest a dual role of CP110 *in vivo* [61]. Germline loss of *Cp110* results in neonatal lethality due to severe cilia defects, with phenotypes reminiscent of human short ribpolydactyly syndrome, a form of ciliopathy with significant skeletal abnormalities. In *Cp110-*null embryos, primary cilia formation is compromised in multiple tissues with impaired Shh signaling, indicating a positive role of CP110 in ciliogenesis. Indeed, CP110 is required for docking of the BB to plasma membrane in early stages of cilia formation. CP110 loss results in abnormal distribution of SDA components. In *Cp110* knockout mouse embryonic fibroblasts (MEFs) generated from these embryos, both SDAs and DAs are absent in the majority of the BB and development of the TZ is compromised [61]. Thus, while eventual removal of CP110 from the distal end of the MC is a prerequisite for cilia growth, CP110 also plays a positive role during earlier steps of ciliogenesis.

### 5.2. CEP290

CEP290 is localized to the centrosomes in dividing cells and distributes to the distal MC in quiescent cells and the TZ in primary and sensory cilia, in which CEP290 serves as a hub to connect the MKS and NPHP complexes. CEP290 interacts with a number of ciliary proteins including: RPGR, RPGR-interacting protein 1 (RPGRIP1), dynactin subunits, kinesin-2 subunits KIF3A and KAP3, CETN1, PCM-1, Ninein, NPHP5, CP110, and CC2D2A [166,168,169]. *CEP290* mutations can result in a broad spectrum of clinical manifestations, including LCA, JS, JSRD, NPHP, SLS, and MKS [170–172]. LCA is associated with mutations throughout *CEP290*, while JS and MKS mutations are mainly located at the C-terminus and N-terminus, respectively [173,174].

The most common retinopathy (LCA) mutation in *CEP290* gene is an A-to-G nucleotide change in intron 26 (c.2991 + 1655A >G; IVS26) that creates a novel splice donor site and results in a 128-bp cryptic exon with a premature stop codon [175,176]. Aberrant splicing caused by this mutation is more pronounced in human photoreceptors than in other cell types, providing a plausible explanation for a penetrant phenotype in the retina [175]. So far, no clear genotype-phenotype relationship is established for *CEP290*-associated ciliopathies, though the function of residual CEP290 from hypomorphic alleles may be related to the less severe clinical manifestation [177]. *AHI1* and *RPGR* variants are reported to modify phenotypes associated with *CEP290* mutations [178,179].

The CEP290 protein level remains constant throughput the cell cycle, including in the G0 phase, yet pleiotropic functions of CEP290 appear to be blocked by CP110 until the exit from the cell cycle and subsequent removal of CP110 [166]. CEP290 interacts with the centriolar satellite protein PCM-1. The depletion of CEP290 disrupts subcellular distribution and PCM-1 complex formation in cultured cells, leading to disorganization of cytoplasmic microtubule network and disruption of centriole migration and protein trafficking to the centrosomes [37]. Notably, induced pluripotent stem cell (iPSC)-derived photoreceptors from LCA patients and JSRD patient fibroblasts display defects in docking of preciliary vesicles and ciliary membrane formation [181]. Treatment of cells with drugs that inhibit actin filament polymerization and/or actin dynamics, such as cytochalasin D or latrunculin B, alleviates ciliogenesis defects caused by the loss of CEP290 [182,183]. Taken together, these data suggest that CEP290 mutations have an impact on cytoplasmic microtubule and/or actin network. Indeed, the CEP290 myosin tail domain indicates actin-related functions of the protein [172,184].

In *C. reinhardtii* and *C. elegans*, absence of CEP290 does not affect cilia formation but leads to altered ciliary protein composition [185,186], suggesting a gating function of CEP290. In concordance, JSRD patient fibroblasts having no detectable CEP290 protein show elevated Shh signaling [181]. Mice with complete loss of *Cep290* die before weaning due to cilia defects in multiple organs including hydrocephalus [174]. In photoreceptors of *Cep290*^*−/−*^ mice, the BB fails to dock to the apical cell membrane, leading to complete failure of OS morphogenesis. In another study, a mouse line carrying gene-trap in intron 23 of *Cep290* displays a less severe phenotype, and the mice are fertile and viable beyond one year [187]; however, the milder phenotype might be attributed to leakage of gene trap alleles [173]. The *rd16* mouse is a spontaneous *Cep290* mutant with an in-frame deletion in the myosin tail of CEP290, leading to rapidly- progressing degeneration of photoreceptors but no syndromic phenotype [184]; though olfactory and hearing defects were identified in later studies [174,188]. Photoreceptors of *rd16* mice have malformed CC and rudimentary OS structure [174], consistent with ciliary defects of photoreceptors in iPSC-derived retinal organoids from *CEP290*-LCA patients [181,189]. Thus, the *rd16* allele is a hypomorph of *Cep290* and phenocopies human *CEP290*-LCA. These studies demonstrate an essential role of CEP290 in the biogenesis and functions of the CC and OS, and a relatively independent role of different domains of CEP290 in cilia biogenesis. However, the precise function of each domain of CEP290 in cilium assembly and mechanisms of distinct CEP290-ciliopathies require further investigation.

We note that CEP290 is also required for targeting of RAB8B GTPase in connection with the BBSome for initiation of IFT and vesicular trafficking [37,166], suggesting an important role of CEP290 in ciliary growth and trafficking. CEP290 directly interacts with BBS6 and loss of CEP290 impairs the recruitment of BBS4 and BBS8 to the BBSome, leading to defects in cilium assembly [169,190]. Although CEP290 does not directly bind to the IFT machineries, its interaction partner NPHP5 binds to IFT22 and facilitates IFT. Deletion of CEP290 myosin tail domain after CC formation resulted in photoreceptor phenotype similar to BBSome mutants, with mis-localization of rhodopsin in the IS and synapse protein syntaxin 3 and syntaxin-binding protein 1 transported to the OS [191]. Notably, the OS of these mutant photoreceptors do not show rapid degeneration. Given the high turnover rate of photoreceptor OS, CEP290 likely has distinct roles at different stages of cilium biogenesis. Future studies on CEP290 interactome at different developmental stages would provide useful insights into its function in photoreceptors.

### 5.3. CC2D2A

CC2D2A is an interaction partner of CP110, CEP290 and another TZ protein TCTN1 [192], all of which participate in cilium assembly. In humans, *CC2D2A* mutations lead to RP, JS, and MKS [193–195]. *Cc2d2a* mutations appear to manifest species-specific phenotypes. In zebrafish, photoreceptors of *Cc2d2a-*null mutants form shorter OS with mis-localization of opsins and Rab8a-associated vesicles in the IS, suggesting its role in ciliary trafficking [196,197]. In contrast, loss of CC2D2A in mammals leads to more severe phenotypes. Loss of *Cc2d2a* in mice showed embryonic lethality caused by severe abnormalities in multiple tissues due to the absence of cilia, resembling human MKS [192]. The retina of a rare survivor *Cc2d2a* null mouse reveals severe disruption of the outer nuclear layer, where photoreceptor cell bodies are located, with poorly developed IS and OS and absence of ERG responses. Similarly, *Cc2d2a* MEFs show the absence of primary cilia and impaired assembly of SDA at the MC, suggesting an indispensable role of CC2D2A in the formation of SDA in mammalian cells. The underlying mechanism of discrepancy between zebrafish and mammals needs further investigation.

## 6. Innovative technologies and therapies for retinal ciliopathies

No treatment options are currently available for ciliopathies. Most ciliopathies exhibit little genotype-phenotype relationship, showing more complex disease etiologies [13]. Mutations in one gene can be implicated in multiple distinct ciliopathies affecting multiple organs with variable manifestations. For example, mutations at different sites of *CEP290* can lead to non-syndromic LCA, syndromic JS and JSRD, BBS, MKS or SLS. This variance is thought to be related to the residual functional domain(s) of the hypomorphic protein [177]. Yet, it is unclear whether these different ciliopathy-associated mutations affect distinct functions of CEP290 at different locations, or if they form an allelic series impacting the same function [13]. Similarly, different mutations in *CC2D2A* lead to MKS and JS with little phenotypic overlap [198]. Mutations can also alter protein isoforms with distinct functions, such as mutations in ARL6 (BBS3). Disruption of a major *ARL6* (*BBS3*) variant leads to typical syndromic phenotypes, while mutations in a longer isoform are shown to affect photoreceptor survival in zebrafish and mice [199]. Genetic modifiers can also influence clinical features of mutations in ciliopathy-associated genes. For example, RPGRIP1 and RPGRIP1L might have overlapping functions in mammals and phenotypes caused by mutations of one gene may be modified by the other [13]. CEP290 and MKKS both participate in the formation of the TZ, and mutations in either are deleterious for cilia formation, yet a combination of *Cep290*^*rd16*^ and *Mkks*^*ko*^ alleles in mice surprisingly leads to improved ciliogenesis and sensory functions [200]. These findings suggest complex protein interactions in biogenesis and maintenance of the primary cilium and pose significant obstacles in identifying the causal allele for therapies. Moreover, with such a large number of disease-associated ciliary genes, it would be expensive and time-consuming to design and evaluate treatments for individual mutations.

Although biochemical and genetic studies have provided substantial information on the interactome of primary cilia, advances in high resolution imaging technologies are needed to elucidate the organization of ciliary structures and functions. For example, the TZ is supposed to be a hotspot for ciliopathies, yet the detailed structure of the TZ and how it controls ciliary composition is still unclear. The *C. elegans* orthologue of CEP290 localizes to the TZ axoneme and is required for the formation of the central cylinder, while the *Chlamydomonas* CEP290 is located in the Y-links and necessary for their formation [185,201]. We have poor understanding of whether CEP290 protein spans the TZ axoneme and Y-links, or if the locations and functions of CEP290 are species-specific. Clarification of these divergent observations would provide useful insights to elucidate the function of ciliary proteins in cilium biogenesis and pathologies.

Current studies on underlying mechanisms of ciliopathies are based on cell lines and/or animal models. These *in vivo* and *in vitro* models undoubtedly have provided significant insights, yet pathophysiology of ciliopathies in humans is still poorly understood. For example, the most common mutation in *CEP290*-LCA patients is an intronic mutation (c.2991 + 1655A >G) that leads to inclusion of a cryptic exon with a stop codon [176], but efforts to replicate this allele precisely in mice have not been successful [202]. Also, different mutations in the same gene may lead to different phenotypes in different tissues. For instance, hypomorphic mutations in the core IFT-B complex protein IFT172 lead to skeletal defects, while other mutations in the same gene cause retinal degeneration. These observations suggest cell type-specific functions of ciliary proteins or distinct mechanisms of disease pathogenesis in different tissues [203]. However, some cell types such as photoreceptors are difficult to maintain in primary cultures [204]. While cell lines offer certain advantages, they do not mimic cell type-specific features or tissue-specific microenvironments to investigate disease progression; such is the case with photoreceptors, a highly polarized cell type with unique morphological and functional features.

Application of new imaging modalities has begun to unravel unprecedented resolution of the primary cilium and uncover previously unappreciated features of this structure. High-throughput and high-content screening coupled with CRISPR-based genome editing has enabled a deeper investigation of genes associated with specific features of the primary cilium in a global and highly efficient manner. With the innovation of three-dimensional culture systems, human PSCs can be coaxed into retinal organoids under proper developmental cues in an appropriate spatiotemporal context. These organoids develop all major retinal cell types capable of self-patterning into a laminated structure with a rudimentary OS-like structure in photoreceptors, offering a tissue-relevant cell source to study photoreceptor OS ciliogenesis and modeling ciliopathies [205,206]. More importantly, iPSCs-derived retinal organoids from ciliopathy patients show disease-associated ciliary defects, providing a valuable *in vitro* platform to study disease pathology and develop therapies [181,207–209]. In the following section, we will review recent advances and applications of imaging modalities, gene therapy and drug discovery in understanding disease mechanisms and developing treatments for ciliopathies.

### 6.1. Imaging modalities for the ciliary structure

TEM is traditionally the most widely used tool for examining the ultrastructure of cilia in healthy and diseased states. The wavelength of an electron beam is 100,000-fold shorter than that of light in the visible range (400 nm for blue light). As spatial resolution is diffraction limited and inversely proportional to wavelength, this explains the superior resolution of TEM compared to light microscopy [210]. However, one limitation of TEM is that it does not reveal the identity of ciliary proteins, thus providing little information on the architecture of compact multiprotein complexes such as the TZ. Immuno-EM was developed to overcome this obstacle and has been applied to reveal the localization of TZ proteins and the docking site of IFT machineries [185,211], yet the sample preparation process is technically challenging and time-consuming. Stimulated emission depletion imaging (STED) microscopy is an optical physics-based imaging technique, which is able to reveal the relative locations of protein complexes without tedious sample processing. It captures the signals of various fluorophores from collective samples and recreates the relative localization of ciliary proteins using position averages, which are then overlapped with representative TEM images [212]. This technique has provided new insights into the architecture of the TZ and may reveal novel functions of well-known ciliary proteins. For example, the distribution of IFT complex components were found to be associated with the cilium growth condition and mainly accumulated at the transition fibers and the distal end of the TZ, suggesting unexplored mechanisms of IFT in cilium biogenesis.

One significant limitation of TEM is that the thin sections do not represent the three-dimensional architecture of the cilium. Although serial section-TEM can partially achieve this goal, it is time-consuming, labor-intensive, prone to artifacts and has low resolution along the axis perpendicular to the cutting plane. There was no satisfactory technology that can efficiently image an entire cell in three dimensions until the advances of focused ion beam scanning electron microscopy (FIB-SEM). FIB-SEM incorporates etches by a beam of focused ions and scanning electron microscope (SEM) to achieve consistent z slices with a thickness of 3 nm [213]. Combination of FIB-SEM with correlative light and electron microscopy enables the detection of ciliary structures with associated membrane tubules, and EHD1+ membrane tubules are shown to be connected to the ciliary pocket membrane to facilitate the development of primary cilia [54].

Recent development in Cryo-EM has permitted sample preparation in near native conditions, allowing direct observation of multiple conformations at atomic resolution [214]. A recent application of Cryo-EM on *T. thermophila* reveals new classes of ciliary proteins that are associated with the microtubule doublets and may have a function in stabilizing the structure and facilitating the elongation of β-tubule [25]. However, information on the connection between the ciliary axoneme and the BB is often lost upon sample processing. Cryo-electron tomography (cryo-ET), which utilizes a thicker sample combined with subvolume averaging, can maintain all relevant spatial information, albeit at the expense of resolution and potential for averaging [215]. This technique has revealed previously undiscovered details of the BB and the TZ, including the two structurally distinct regions along the proximal-distal axis in mammalian centrioles, as well as the photoreceptor-specific extension of the distal CC [35,128]. A few studies have coupled cryo-ET with super-resolution stochastic optical reconstruction microscopy (STORM) to define the subdomain structure of the TZ. STORM uses antibodies conjugated to specialized fluorophores, whose emission can be captured by super-resolution fluorescence nanoscopy in order to localize specific proteins at resolutions well below the diffraction limit of immunofluorescence [216]. This new advance has successfully identified key molecular signatures in the TZ and provided new insights in BBS and JS disease mechanisms [68,217]. These advances in imaging modalities would be useful to link specific mutations to alterations of ciliary structure, facilitating the structure-based drug design [218].

### 6.2. Gene therapy

Gene therapy by gene replacement or gene editing has been successful in several mammalian ciliopathy models and can potentially rescue ciliary defects [219]. Delivery of a full-length or partial wild-type gene would be a useful approach for treatment of ciliopathies. Delivery of a full-length wild-type gene into relevant disease tissues can restore cilia structures and functions in animal models [219]. However, questions remain as to whether there would be undesirable side-effect (s) by overexpression of certain proteins, particularly if the target cells are already under stress due to the disease mutation. In addition, the limited packaging capacity of adeno-associated virus (AAV) (~5 kb), at present the most efficient gene delivery vehicle to retinal neurons, is insufficient to deliver genes with larger sizes into target tissues. An alternative approach is to deliver only a partial gene encoding a specific functional domain impacted by mutation(s). The feasibility of such an approach has been demonstrated recently, where the myosin tail domain of CEP290 is delivered to the degenerating *rd16* retina [220]. The treated *rd16* mice show structural and functional maintenance of photoreceptors, suggesting that the delivered myosin tail domain is able to cooperate with the endogenous hypomorphic CEP290 protein to restore ciliogenesis and photoreceptor function.

With advances in CRISPR-based genome editing approach, ciliopathy-causing mutations can in theory be corrected for therapeutic purposes. In a recent study, a genome-editing cassette was shown to target IVS26 in *CEP290* and restore normal CEP290 expression in a human cell line, retinal explants, humanized mice, and non-human primates with a desirable efficiency [221]. Similarly, CRISPR-based approach has been used to correct frameshift or deletion mutations in ciliopathy patient iPSCs [207,208]. Retinal organoids and/or retinal pigment epithelium differentiated from these corrected patient iPSCs demonstrate improved morphology of cilia structure and gene profiles resembling the control transcriptomes compared to the untreated ones. These studies provide important proof-of-concept evidence for the application of CRISPR-based therapeutic approaches in the treatments of ciliopathies. However, the transcriptomes of retinal tissues differentiated from corrected iPSCs display deviations from the control, likely arising from off-target effects of CRISPR-based system. In addition, it is still unclear what the effective treatment window for gene repair would be. Whether morphological and functional rescue of phenotypes could be achieved when patients reach the appropriate age for such treatments require further investigation. Additionally, the single-base editing efficiency of CRISPR-based system is far below therapeutic needs (< 10% in monolayered cell culture) and is not suitable for ciliopathies caused by point mutations at this stage [222].

A transcript repair approach utilizes antisense oligonucleotides (AONs) to restore normal splicing of genes affected by mutations at splicing donor/receptor sites. The AON approach could partially restore normal splicing of IVS26 in treated retinal organoids derived from iPSCs of a *CEP290*-LCA patient, demonstrating improved morphology of photoreceptors [189]. Moreover, no serious adverse effects are observed in a clinical trial with 10 patients treated with this approach, and the patients report some vision improvement [223].

Recent development of high-throughput screening using CRISPR-based gene disruption have provided new paradigms for treatment development. In initial screens, a pool of single-guide RNAs (sgRNAs) was introduced in bulk into mouse fibroblast cell lines engineered with cilium-dependent Hh signaling driven anti-blasticidin reporter gene [224]. In this case, genes affecting ciliary Hh signaling could be identified through their modulation of blasticidin resistance. Such an approach can be tailored with different reporters using patient fibroblasts or iPSC-derived tissues to identify key ciliary genes to restore appropriate ciliary signaling.

### 6.3. Drug discovery

With over 200 genes identified for ciliopathies and the continuously increasing number of identified causal genes [13], it is not feasible to tailor gene therapy for individual mutations. Therefore, various compounds targeting disease-associated symptoms or cilia biogenesis are developed as an alternative therapeutic approach. For example, primary ciliary dyskinesia can be caused by mutations in genes encoding an axonemal dynein intermediate chain necessary for ciliary motility, and phenotypes include dysfunction in respiratory motile cilia, sperm flagella, and nodal cilia [225]. Antibiotic administration is now used to reduce inflammation and techniques for airway clearance are also applied to maintain the respiratory function [226–228]. In retinal ciliopathies, delaying or even blocking apoptosis of photoreceptor cells can provide a means for maintaining patient vision. Supplementations of antioxidants such as vitamin A, vitamin B3, docosahexaenoic acid (DHA) or lutein have been shown to prevent photoreceptor apoptosis [229,230]. Neurotrophic agents such as ciliary neurotrophic factor, brain-derived neurotrophic factor, and anti-apoptotic drug (tauroursodeoxycholic acid, rasagiline, norgestrel, and myriocin) have been applied in the treatment of retinitis pigmentosa [231]. Pharmaceutical modulations of unfolded protein response caused by ciliary defects using valproic acid, guanabenz, and a specific Caspase 12 inhibitor have also been demonstrated to protect photoreceptors and maintain light detection in *Bbs12*^*−/−*^ animal model [232].

Approximately 10% of human genetic diseases are caused by nonsense mutation-induced premature termination codon (PTC) in the coding region of mRNA, leading to generation of truncated protein with missing or no function(s) [233]. Read-through drugs that are able to overcome PTC in translation offer a promising approach to restore protein function(s) and reduce disease symptoms without editing genome or transcriptome of patients. Treatment of spinal muscular atrophy patient fibroblasts and animal models with pyranmycin TC007 has shown substantial increase of full-length protein in both systems, as well as longer survival of motor neurons and longer lifespan of the mice, suggesting the feasibility of such a pharmaceutical approach [234,235]. Another read-through drug PTC124 also demonstrates biocompatibility and recovery of protein functions in cell culture, retinal explant and animal models of Usher 1C [236]. While the applications of read-through drugs on animal models have shown positive results, much remains to be done. Major safety and technical concerns including toxicity, bioavailability, efficacy must be solved before clinical use of these drugs.

A promising study of high-throughput screening on *CEP290*^*−/−*^ RPE1 cell line has identified Eupatilin as an effective therapeutic agent to rescue defects in the TZ caused by *CEP290* mutations through an increased recruitment of NPHP5 protein to the TZ [237]. Intravitreal injection of Eupatilin into *rd16* mice, the animal model of *CEP290*-LCA, reveals maintenance of cone photoreceptors and a modest functional recovery. This study demonstrates the feasibility of treatment of ciliopathies by modulation of ciliary proteins by small molecule drug(s). However, rod photoreceptors, which are the first cell type impacted by the disease, cannot be maintained by Eupatilin. This could be due to faster degeneration of rod photoreceptors, or perhaps that rod photoreceptors utilize an alternative mechanism for cilium biogenesis compared to RPE1 cells. Although cilium biogenesis and ciliopathies demonstrate cell type-/tissue-specific features, performing high-throughput screening using mouse or human photoreceptors is technically challenging, due to small number of cells in the retina. Alternatively, stem cell-derived retinal organoids can be used to generate photoreceptor cells in sufficient quantity [238]. Mouse retinal organoids have a short differentiation time (< 35 days) and high differentiation efficiency, making them suitable for large-scale screening [239]. Although a better model to recapitulate human pathology, the retinal organoids generated from patient iPSCs take as much as 180 days for differentiation and requires labor-intensive manipulations [206], posing significant challenges for large-scale applications such as high-throughput screening. However, a recent protocol bypasses the dissection process and enables simple and efficient large-scale production of human retinal organoids to meet the high demand of cells in the screening [240]. A pharmaceutical approach is recently reported to convert mouse fibroblasts directly to photoreceptors by five small molecules [241]. Although a desirable approach, the differentiation efficiency and the polarity of two-dimensional photoreceptors require further investigation. For the treatment design of high-throughput screening and use of retinal organoids for treatment, we direct our readers to our recent reviews [242,243].

## 7. Conclusion

In conclusion, the biogenesis of primary cilium requires orchestrated actions of numerous complex protein networks. Mutations disrupting these networks lead to structural and/or functional defects of the primary cilium, which are manifested as a broad spectrum of diseases called ciliopathies. Photoreceptors harbor a specialized primary cilium with unique features, which make it especially vulnerable to functional defects. The accessibility to retinal photoreceptor cilia offers a unique opportunity to investigate disease mechanisms and evaluate treatments for ciliopathies. Recent innovation in technologies should accelerate this process.

## Figures and Tables

**Table 1:** Functions of major disease-associated proteins in cilia biogenesis [13,18,20,124,150].

Gene symbol	Protein	Function	Associated disease(s) in humans or mice
Centriole and pericentriolar material (PCM)			
*PCM1*	Pericentriolar material 1	Centriolar satellite component, anchors microtubules to the centrosome	Papillary thyroid carcinoma in humans; reduced brain size in haploinsufficient mice
*PCNT*	Pericentrin	Pericentriolar material component, important to normal functioning of the centrosomes, cytoskeleton, and cell-cycle progression	Seckel syndrome-4 and microcephalic osteodysplastic primordial dwarfism-2 in humans
Daughter centriole			
*CEP120*	Centrosomal protein 120	Microtubule-dependent coupling of the nucleus and the centrosome	JS-31 and Short-rib thoracic dysplasia-13 in humans
*CNTROB*	Centrobin	Centriole duplication and cytokinesis	Defects in reproduction
Mother centriole			
*OFD1*	OFD1 Centriole and centriolar satellite protein	Centriole and centriolar satellite protein	OFD-1, Simpson-Golabi-Behmel syndrome-2 JS-10, and RP-23 in humans
*C2CD3*	C2 domain-containing protein 3	Centriolar distal appendage assembly;Recruitment of other ciliary proteins, including intraflagellar transport proteins	OFD-14 in humans
Distal appendage			
*CEP83*	Centrosomal protein 83	Primary cilium assembly	NPHP-18 in humans
*SCLT1*	Sodium channel and clathrin linker 1	Cilium assembly	PKD
*CEP164*	Centrosomal protein 164	Microtubule organization, DNA damage response, chromosome segregation and assembly of primary cilia	NPHP-15 in humans
Subdistal appendage			
*TUBG1*	Tubulin gamma 1	Structural component of centrioles, binding to microtubules through interaction with γ-tubulin ring complex of PCM	Complex cortical dysplasia with other brain malformations-4
*CP110*	Centriolar coiled-coil protein 110	Regulator of cell cycle; Inhibitor of ciliogenesis	Defects in multi-organ development
*CC2D2A*	Coiled-coil and C2 domain containing 2A	Cilium formation	MKS and Visceral heterotaxy in mice; MKS-6, JS-9 and COACH syndrome in humans
*ODF2*	Outer dense fiber of sperm tails 2	Major outer dense fiber protein	Infertility
*NIN*	Ninein	Positioning and anchoring the microtubules minus-ends	Seckel syndrome-7 in humans
Motor protein			
*KIF3A* *KIF3B*	Kinesin family member 3A Kinesin family member 3B	Component of heterotrimeric kinesin-2 motor complex	PKD-1; dysostosis Mice with null mutation die during the midgestational period
*KIFAP3*	Kinesin associated protein 3		70% homozygous knock-out die from heart failure shortly after birth
*KIF17*	Kinesin family member 17	Component of homodimeric kinesin-2 motor complex	Impaired neural functioning
*DYNC2H1*	Dynein cytoplasmic 2 heavy chain 1	Component of cytoplasmic dynein 2	Asphyxiating thoracic dystrophy 3 in both humans and miceVACTERL association in mice
*WDR34*	WD repeat domain 34		Short-rib thoracic dysplasia-11 with or without polydactyly
*DYNLL1*	Dynein light chain LC8-type 1		Preweaning lethality in mice
*DYNC2LI1*	Dynein cytoplasmic 2 light intermediate chain 1	Retrograde transport of cargo in primary cilia via the intraflagellar transport system	Short-rib thoracic dysplasia-15 with polydactyly
Transition zone (TZ)			
*TCTN1*	Tectonic family member 1	Component of the MKS complex	JS-3 in humans
*TCTN2*	Tectonic family member 2		MKS-8 and JS-24 in humans
*TCTN3*	Tectonic family member 3		OFD-5 and JS-18 in humans
*MKS1*	MKS transition zone complex subunit 1		MKS-1, JS-28 and BBS-13 in humans; MKS, atrioventricular septal defect and tetralogy of Fallot in mice
*B9D1*	B9 domain containing 1		JS-27 in humans; MKS in mice
*B9D2*	B9 domain containing 2		Ciliogenesis defects in humans; preweaning lethality in mice
*CC2D2A*	Coiled-coil and C2 domain containing 2A		MKS-6, JS-9 and COACH syndrome in humans; MKS and Visceral heterotaxy in mice
*TMEM67*	Transmembrane protein 67		NPHP-11, MKS-3, COACH syndrome, and BBS-4 in humans; MKS, cystic kidney disease, and visceral heterotaxy in mice; JS-6 in both humans and mice
*TMEM216*	Transmembrane protein 216		JS-2 and MKS-2 in humans
*TMEM231*	Transmembrane protein 231		JS-20 in humans
*TMEM107*	Transmembrane protein 107		MKS-13 and OFD-15 in humans
*CEP290*	Centrosomal protein 290	Protein hub of the MKS and NPHP complexes	BBS-14, MKS-4, and SLS in humans; Cystic kidney disease and
*NPHP1*	Nephrocystin 1	Component of the NPHP complex	SLS, NPHP-1, and JS-4 in humans
*NPHP3*	Nephrocystin 3		Renal-hepatic-pancreatic dysplasia and MKS-7 in humans; PKD in mice; NPHP-3 in both humans and mice
*NPHP4*	Nephrocystin 4		SLS and NPHP-4 in humans; NPHP in mice
*IQCB1(also called NPHP5)*	IQ motif containing B1		SLS and LCA in humans
*NEK8*	NIMA related kinase 8		NPHP-9 and renal-hepatic-pancreatic dysplasia in humans; PKD in mice
*ANKS6*	Ankyrin repeat and sterile alpha motif domain containing 6		NPHP-16 in humans; visceral heterotaxy in mice
*INVS*	Inversin	Component of the NPHP complex, protein hub of the MKS and NPHP complexes	NPHP-2 in both humans and mice; Tetralogy of Fallot in mice
*INPP5E*	Inositol polyphosphate-5phosphatase E	Regulate Golgi-vesicular and cilia-vesicular trafficking	JS-1 in humans
*RPGR*	Retinitis pigmentosa GTPase regulator	Guanine nucleotide exchange factors interacts with RPGRIP1	RP-3 and X-linked cone-rod dystrophy-1 in both humans and mice
*RPGRIP1*	RPGR interacting protein 1	Interacts RPGR to facilitate ciliogenesis	LCA-6 in both humans and mice; Cone-rod dystrophy-13 in humans
*RPGRIP1L*	RPGRIP1 like	Interacts with interact with NPHP4	MKS-5 and COACH syndrome in humans; MKS in mice; JS-7 in both humans and mice
Intraflagellar transport (IFT)			
*IFT20, 46, 52, 57*	Intraflagellar transport 20, 46, 52, 57	Component of IFT complex B	Homozygous null mutation embryonically lethal in mice
*HSPB11*	heat shock protein family B (small) member 11		Homozygous null mutation neonatally lethal in mice
*IFT27*	Intraflagellar transport 27		Homozygous null mutation neonatally lethal in mice
*TRAF3IP1*	TRAF3 interacting protein 1		SLS-9 in humans; Homozygous null mutation embryonically lethal in mice
*IFT74*	Intraflagellar transport 74		Visceral heterotaxy in mice
*IFT80*	Intraflagellar transport 80		Asphyxiating thoracic dystrophy-2 in humans and mice; Asphyxiating thoracic dystrophy-3 in mice
*IFT81*	Intraflagellar transport 81		Short-rib thoracic dysplasia 19 with or without polydactyly in humans
*IFT88*	Intraflagellar transport 88		PKD in mice
*IFT172*	Intraflagellar transport 172		RP-71 and short-rib thoracic dysplasia-10 with or without polydactyly in humans; Retinal degeneration and atrioventricular septal defect in mice
*IFT43*	Intraflagellar transport 43	Component of IFT complex A	Cranioectodermal dysplasia-3 in humans
*WDR35*	WD repeat domain 35		Sensenbrenner syndrome in humans; Short-rib thoracic dysplasia 7 with or without polydactyly in both humans and mice
*TTC21B*	Tetratricopeptide repeat domain 21B		NPHP-12 and asphyxiating thoracic dystrophy-4 in humans; Cystic kidney disease in mice
*IFT140*	Intraflagellar transport 140		Short-rib thoracic dysplasia 9 with or without polydactyly in both humans and mice; Asphyxiating thoracic dystrophy-1 in mice
*WDR19*	WD repeat domain 19		NPHP-3, craniometaphyseal dysplasia, and asphyxiating thoracic dystrophy 5 in humans; ciliopathy in mice
*TULP3*	TUB like protein 3	Cargo adaptor for IFT complex A	PKD in mice
*CLUAP1*	Clusterin associated protein	Cargo adaptor for IFT complex B	Homozygous mutant mid-gestationally lethal in mice
*TTC26*	Tetratricopeptide repeat domain 26		Homozygous for a spontaneous nonsense partially prenatally lethal in mice
*BBS1, 4*	BBS1, 4	Component of the BBSome	BBS in both humans and mice; Obesity in mice
*BBS2*	BBS2		BBS-2 in both humans and mice; RP-74 in humans
*BBS5, 7, 9*	BBS5, 7		BBS humans
*TTC8*	Tetratricopeptide repeat domain 8		BBS-8 in both humans and mice; RP-51 in humans
*BBS10*	BBS10	Interacts with the BBSome; A molecular chaperone that may affect the stability and folding of other ciliary proteins	BBS-10 in humans
*UNC119* *PDE6D*	Unc-119 lipid binding chaperone Phosphodiesterase 6D	Lipid-binding transporter proteins to facilitate the transport of ciliary periphery membranes across the TZ	Retinal degeneration in homozygous null mice JS-22 in humans
Photoreceptor-specific outer segments (modified primary cilium)			
*PRPH2*	Peripherin 2	Structural outer segment proteins	RP-7 in both humans and mice; fundus albipunctatus, partial central choroid dystrophy, patterned macular dystrophy 1, and vitelliform macular dystrophy in humans
*ROM1*	Retinal outer segment membrane protein 1		RP-7 in humans
*TULP1*	TUB like protein 1		RP-14 in both humans and mice; LCA-15 in humans
*CDHR1*	Cadherin related family member 1		CRD-15 in humans
*EYS*	Eyes shut homolog		RP in humans
*FSCN2*	Fascin actin-bundling protein 2, retinal		RP-30 in both humans and mice; auditory system disease and retinal degeneration in mice
*PROM1*	Prominin 1		RP-41 in both humans and mice; CRD and Stargardt disease in humans
*RHO*	Rhodopsin	Photoreceptor sensory function proteins	RP-4 and congenital stationary night blindness autosomal dominant 1 in both humans and mice; fundus albipunctatus in humans
*ABCA4*	ATP binding cassette subfamily A member 4		CRD-3, RP-19 and Stargardt disease in both humans and mice; AMD-2 in humans
*CNGA1*	Cyclic nucleotide gated channel subunit alpha 1		RP-49 in humans
*CNGA3*	Cyclic nucleotide gated channel subunit alpha 3		Achromatopsia 2 in humans
*CNGB1*	Cyclic nucleotide gated channel subunit beta 1		RP-45 in both humans and mice
*CNGB3*	Cyclic nucleotide gated channel subunit beta 3		Achromatopsia 2 in both humans and mice; Stargardt disease in humans
*GNAT1*	G protein subunit alpha transducin 1		Congenital stationary night blindness in humans
*GNAT2*	G protein subunit alpha transducin 2		Achromatopsia 4 in both humans and mice
*GUCA1A*	Guanylate cyclase activator 1A		CRD-14
*GUCA1B*	Guanylate cyclase activator 1B		RP-48 in humans
*OPN1LW*	Opsin 1, long wave sensitive		Partial, protanopic colorblindness in humans
*OPN1MW*	Opsin 1, medium wave sensitive		Blue cone monochromacy in both humans and mice; Red color blindness in humans
*OPN1SW*	Opsin 1, short wave sensitive		Blue color blindness in humans
*PDE6A*	Phosphodiesterase 6A		RP-43 in both humans and mice
*PDE6B*	Phosphodiesterase 6B		RP-40 and congenital stationary night blindness autosomal dominant 2 in both humans and mice
*PDE6C*	Phosphodiesterase 6C		Cone dystrophy in humans; Achromatopsia in mice
*PDE6G*	Phosphodiesterase 6G		RP-57 in both humans and mice
*RDH12*	Retinol dehydrogenase 12		LCA-13 in humans
*RGS9 and RGS9BP*	Regulator of G protein signaling 9 and binding protein		Bradyopsia in humans
*SAG*	S-antigen visual arrestin		RP-47 and Oguchi disease-1 in humans
*ARL6 (BBS3)* *CLRN1*	ADP-ribosylation factor-like 6 Clarin 1	Connecting cilium and axoneme-associated proteins	BBS-3 and RP-55 in humans Usher syndrome type 3A in both humans and mice; RP-61 in humans
*FAM161A*	FAM161 centrosomal protein A		RP-28 in both humans and mice
*KIZ*	Kizuna centrosomal protein		RP-69 in both humans
*MAK*	Male germ cell associated kinase		RP-62 in both humans
*RAB28*	RAB28, member RAS oncogene family		CRD-18 in both humans and mice
*RP1*	RP1 axonemal microtubule associated		RP-1 in both humans and mice
*RP1L1*	RP1 like 1		Occult macular dystrophy in humans
*RP2*	RP2 activator of ARL3 GTPase		RP-2 in both humans and mice
*SPATA7*	Spermatogenesis associated 7		LCA-3 in both humans and mice
*TOPORS*	TOP1 binding arginine/serine rich protein, E3 ubiquitin ligase		RP-31 in humans
*USH2A*	Usherin		Usher syndrome type 2A in both humans and mice; RP-39 in humans

JS, Joubert syndrome; RP, Retinitis pigmentosa; OFD, Orofaciodigital syndrome; NPHP, Nephronophthisis; MKS, Meckel syndrome; PKD, Polycystic kidney disease; BBS, Bardet–Biedl syndrome; SLS, Senior-Løken syndrome; CRD, cone-rod dystrophy; AMD; age-related macular degeneration.

## Abbreviations

AAV: adeno-associated virus
AON: antisense oligonucleotides
AV: adeno virus
BB: basal body
BBS: Bardet-Biedl syndrome
CC: connecting cilium
CLS: ciliary localization sequence
Cryo-ET: cryo-electron tomography
CV: ciliary vesicle
DA: distal appendage
DC: daughter centriole
FIB-SEM: focused ion beam scanning electron microscopy
GPCR: G protein-coupled receptor
Hh: Hedgehog
IFT: intraflagellar transport
iPSC: induced pluripotent stem cells
IS: inner segment
JS: Joubert syndrome
JSRD: Joubert syndrome and related disorders
LCA: Leber congenital amaurosis
MC: mother centriol
MEF: mouse embryonic fibroblast
MKS: Meckel syndrome
mTOR: mammalian target of Rapamycin
NPHP: nephronophthisis
OMIM: Online Mendelian Inheritance in Man database
OS: outer segment
PCM: pericentriolar material
PCD: primary ciliary dyskinesia
PCV: preciliary vesicle
PDGFR: platelet-derived growth factor receptor
PTC: Premature termination codon
RP: retinitis pigmentosa
RPE: retinal pigment epithelia
SDA: subdistal appendage
SEM: scanning electron microscopy
sgRNAs: single-guide RNAs
SLS: Senior-Løken syndrome
STED: stimulated emission depletion imaging
STORM: super-resolution stochastic optical reconstruction microscopy
TEM: transmission electron microscopy
TF: transition fiber
TGF: transforming growth factor
TZ: transition zone
Wnt: Wingless
